# The Neuroprotective Role of Acupuncture and Activation of the BDNF Signaling Pathway

**DOI:** 10.3390/ijms15023234

**Published:** 2014-02-21

**Authors:** Dong Lin, Ike De La Pena, Lili Lin, Shu-Feng Zhou, Cesar V. Borlongan, Chuanhai Cao

**Affiliations:** 1 College of acupuncture, Fujian University of Traditional Chinese Medicine, Minhou Shangjie, Fuzhou 350122, Fujian, China; E-Mails: bluelike1@sina.com (D.L.); linlili77fz@163.com (L.L.); 2 Department of Neurosurgery and Brain Repair, College of Medicine, University of South Florida, Tampa, FL 33612, USA; E-Mails: iked@health.usf.edu (I.D.L.P.); cborlong@health.usf.edu (C.V.B.); 3 College of pharmacy, Department of pharmaceutical Sciences, University of South Florida, Tampa, FL 33612, USA

**Keywords:** acupuncture, brain derived neurotrophic factor, neuroprotective, cyclophilin A

## Abstract

Recent studies have been conducted to examine the neuroprotective effects of acupuncture in many neurological disorders. Although the neuroprotective effects of acupuncture has been linked to changes in signaling pathways, accumulating evidence suggest the participation of endogenous biological mediators, such as the neurotrophin (NT) family of proteins, specifically, the brain derived neurotrophic factor (BDNF). Accordingly, acupuncture can inhibit neurodegeneration via expression and activation of BDNF. Moreover, recent studies have reported that acupuncture can increase ATP levels at local stimulated points. We have also demonstrated that acupuncture could activate monocytes and increase the expression of BDNF via the stimulation of ATP. The purpose of this article is to review the recent findings and ongoing studies on the neuroprotective roles of acupuncture and therapeutic implications of acupuncture-induced activation of BDNF and its signaling pathway.

## 1. Introduction

Acupuncture, which has been used clinically for more than 2500 years in East Asia, has been reported as an effective treatment approach in various kinds of neurological diseases including movement disorders, Parkinson’s disease (PD), and Alzheimer’s disease (AD) [1]. In the theory of Traditional Chinese medicine (TCM), the function of the acupuncture is related to insertion of needles at specific points of the body, referred to as “acupoints”. Insertion of needles at specific acupoints produces functional specificity [2,3]. Recent clinical studies have shown therapeutic benefits of manual acupuncture or electroacupuncture (EA) in the central nervous system (CNS) [4]. Regardless of the method, the needles inserted into acupoints produce special physical stimulation that can facilitate homeostasis.

Moreover, several recent studies have demonstrated neuroprotective activities of acupuncture therapy. A majority of these studies have proposed that acupuncture exerts its therapeutic effects via regulation of various signaling pathways. For instance, it has been found that acupuncture-mediated neuroprotection can decrease p38MAPK levels and reduce the expression of pro-inflammatory cytokines/mediators and pro nerve growth factor (NGF) [5–7], which are involved in apoptotic cell death of neurons and oligodendrocytes [8–11]. In contrast, there are but a few studies which implicated the role of neurotrophins (e.g., BDNF, GDNF) in acupuncture-induced neuroprotection. Brain-derived neurotrophic factor (BDNF), a neurotrophin that regulates the development, regeneration, survival and maintenance of neurons, plays pivotal roles in many aspects of brain function [12]. The most recent data on the correlation between neurotrophins and acupuncture have shown that EA may relieve certain neuropathological disorders by modulating BDNF and its signaling pathway. The beneficial effects of acupuncture have been associated with the release of neuropeptides from nerve endings and modulation of the expression of neurotrophins [13,14]. The purpose of this article is to review the recent findings and ongoing studies on the neuroprotective roles of acupuncture and therapeutic implications of acupuncture-induced activation of BDNF and its signaling pathway.

## 2. Several Kinds of Physical Stimulation Can Activate the BDNF Signaling Pathway

To date, it is widely accepted that different forms of sensory stimulation induce changes in BDNF expression in relevant CNS structures (e.g., light-induced changes in BDNF levels in the visual cortex) [15]. Previous works also demonstrated that physical exercise and environmental enrichment, or dietary restriction, can induce BDNF expression in the brain [16]. Some data revealed that cellular stress resistance induced by physical and mental exercise could activate several kinases and transcription factors that induce the expression of genes which encode proteins that promote cell survival and synaptic plasticity (e.g., BDNF).

Repetitive transcranial magnetic stimulation (rTMS) has been known to change the expression of BDNF. Some researchers demonstrated that rTMS can upregulate neurotrophic/growth factors in 6-hydroxydopamine (OHDA)-lesioned PD condition, and that BDNF is one of the most important neuroprotective proteins expressed after rTMS [17]. Furthermore, high-frequency rTMS can increase *in vivo* BDNF levels in patients with neuropsychiatric disorders. Therefore, the neurotrophic factors induced by rTMS, such as BDNF, may modulate the nigrostriatal DA system, and lead to functional recovery [18,19].

Some researchers have also shown that other kinds of stimulation, such as physical exercise, can enhance neuroprotection. Rats subjected to cerebral ischemia reperfusion (IR) injury showed motor function recovery after physical exercise which exerted neuroprotective activities [20,21]. Other studies have shown that rotarod exercise combined with *S*-nitrosoglutathione (GSNO) can stimulate the expression of neurorepair mediators, such as BDNF and its receptor, tropomyosin receptor kinase B (TrkB) in a stroke rat model induced by middle cerebral artery occlusion. These studies imply that BDNF is required for functional recovery following stroke, because other findings have shown that the beneficial effects of rehabilitation on recovery can be negated by treatment with antisense BDNF oligonucleotide [22,23]. Furthermore, some evidence indicated that treatment with phosphoinositide-13 (PI3) kinase inhibitor can reverse the beneficial effects of exercise in neurorepair. This means that the neuroprotective effects of BDNF may be linked with the activation of the PI3 kinase/Akt pathway to accelerate the recovery of neurological exercise and activate phosphatidylinositol [24].

Meanwhile, many types of electrical stimulation (ES) devices have been shown to promote the survival of degenerated neural cells via increasing the levels of BDNF proteins [25]. A growing amount of experimental evidence suggests that ES could not only directly upregulate the transcriptional induction of BDNF in glial cells, but also induce the production of endogenous BDNF from Müller cells. Accordingly, BDNF may inversely regulate the anti-inflammatory response by attenuating microglial activation [26,27]. In addition, other evidence demonstrated that anti-BDNF neutralizing antibody significantly inhibited the activity of Müller cell to rescue light-damaged photoreceptors. Thus, BDNF might act as an important molecule that could facilitate the survival of light-exposed photoreceptor cells via ES. As for its mechanism, some researchers believe that BDNF-mediated cell survival may be attributed to two types of transmembrane glycoproteins, the high-affinity tyrosine kinase receptors (Trk) and the low-affinity neurotrophin receptor p75 (p75NTR) [28].

## 3. The Neuroprotective Roles of BDNF/TrkB Signaling

BDNF protein is widely distributed in the neuronal cell bodies, axons and dendrites of the CNS, and is also widely involved in neural plasticity important for learning and memory [29,30]. It is well known that some neuronal activities such as electrical-evoked stimulation also regulate the transport of BDNF mRNA and proteins into dendrites. These mechanisms are considered to be responsible for the ability of locally translated BDNF to modulate synaptic transmission and synaptogenesis [31]. Moreover, BDNF can act via autocrine and paracrine mechanisms, depending on the site of cell surface receptors through which it signals [32]. The binding of BDNF to TrkB can initiate various intracellular signaling pathways, including mitogen activated protein kinase/extracellular signal-regulated protein kinase (MAPK/ERK), phospholipase Cg (PLCγ), and phosphoinositide 3-kinase (PI3K) pathways [33,34]. Several reports showed that BDNF prevented cell death caused by DNA damage by activating the ERK pathway in cortical neurons and cerebellar neurons. Other research reports indicated that the protective effect of BDNF against apoptosis not only requires the activation of the PI-3K/Akt, but also activate ERK pathways through inhibition of GSK-3β and activation of *cAMP response element-binding* protein (CREB). It has been observed that BDNF also can protect cortical neurons against camptothecin- or serum deprivation-induced apoptosis through activation of ERK and PI3 kinase pathways [35]. As for the mechanism of BDNF-induced protection against neuronal apoptosis, some research have demonstrated that BDNF prevents neuronal death caused by N-methyl-D-aspartate receptor (NMDAR) blockade in corticostriatal organotypic cultures, and its effects are dependent on stimulation of the ERK and PI-3K/Akt signaling cascades [36].

## 4. The Neuroprotective Effects of Acupuncture in Brain Function

The beneficial effects of acupuncture have been largely attributed to sensory stimulation [37]. Needle insertion into the skin and deeper tissues results in particular patterns of afferent activity in peripheral nerves. The inserted needles are stimulated by manual rotation or through the application of ES, generally referred to as EA [38]. Identification of β-endorphin as the factor mediating the pain-relieving effects of acupuncture, the central mechanism of acupuncture, represents a milestone in the history of acupuncture research [39]. However, many other molecules and systems may also be stimulated by acupuncture resulting in a number of biological effects, such as modulation of stress, pain, autonomic activity and immune systems.

Research progress in the last 20 years has shown that it is possible to affect synthesis of BDNF contributing to the restoration of normal systemic balance through acupuncture. Currently, acupuncture is a relevant therapy in complementary and alternative medicine, and it is believed to release and influence the action of several neurotransmitters (e.g., glutamate, acetylcholine, GABA, and serotonin), and neuropeptides in both CNS and peripheral nervous systems (PNS), respectively [3,40,41].

Scientists have tried to understand how puncture at certain acupoints can affect the distant and complicated system such as the CNS [42]. Previous studies suggested that acupuncture acted as a neuromodulating input into the CNS [43]. Recent research showed that acupunctural signal could travel to the CNS via afferent nerve pathways and cause various neurological and physiological changes [44]. For example, Choi *et al*. suggested that acupuncture stimulation is responsible for the protection of neurons by accelerating cerebral blood flow and also increasing plasma osmolality and extracellular glutamate in diabetic rats under ischemic conditions [7]. Other research showed that acupuncture can inhibit the nigrostriatal neurodegeneration caused by 1-methyl-4-phenyl-1,2,3,6-tetrahydropyridine (MPTP) intoxication by increasing expression levels of tyrosine hydroxylase (TH) and dopamine transporter (DAT). Moreover, acupuncture at the GB34 and LR3 acupoints, compared with non-acupoints in the hip, in an MPTP model led to significantly higher levels of TH cells in the striatal and substantia nigra pars compacta regions in mouse brain tissue [5]. Acupuncture treatments in animal experiments have generated valuable mechanistic insights into the pathology of PD and have provided evidence that acupuncture therapy is neuroprotective and can increase various neuroprotective agents such as BDNF, GDNF, and cyclophilin A (CypA) [45].

## 5. Acupuncture Therapy and BDNF Signaling Pathway in Various Disease States

As discussed above, acupuncture can serve as important therapeutic intervention due to its multiple neuromodulatory functions in the CNS. Recently, accumulating data (Table 1) have shown that acupuncture exerts several beneficial effects in the CNS via activation of BDNF and its down-stream signaling pathway [46,47].

### 5.1. Depression

Manual and EA stimulation at zusanli (ST36) produced a variety of neuromodulatory functions in the CNS, especially in the hippocampus of rats exposed to immobilization stress. EA stimulation can notably augment the expression of BDNF mRNA, and also BDNF mRNA levels in the hippocampus of AD patients [48]. As for the mechanism of acupuncture in depression, it was reported that EA and manual acupuncture can upregulate expression of hippocampus BDNF protein and mRNA as well as its receptor TrkB. Furthermore, this effect may be explained by activation of adenyl cyclase-cAMP-PKA-CREB signaling pathway [49]. Acupuncture at “Baihui” (GV 20) and “Yintang” (EX-HN 3) can effectively reverse chronic stress-induced down-regulation of BDNF mRNA and protein expression in the frontal cortex and hippocampus of rat models of depression, contributing to its anti-depressive-like actions by protecting neuronal regeneration [50].

### 5.2. Cerebral Ischemia Injury

EA treatment has the ability to relieve cerebral ischemia-reperfusion injuries and provide neuroprotection by interfering with the expression of multiple apoptosis-related genes, such as c-fos, heat shock protein 70 (HSP70), and even the neurotrophin (NT) family of proteins [51–53]. For example, acupuncture can exert beneficial effects in the CNS by enhancing BDNF mRNA expression levels in the hippocampus of mice following cerebral ischemia-reperfusion injury, which protects neurons from injuries, and inhibits apoptosis of hippocampal cells. At the same time, EA treatment at the acupoint of Baihui GV(20) significantly increased expression of BDNF and TrkB and improved motor recovery [54]. Some researchers believe that acupuncture can exert neuroprotective function in ischemic stroke via activation of the PI3K/Akt pathway [55]. Furthermore, EA pretreatment can enhance the tolerance to focal cerebral ischemia, via upregulation of BDNF and the chemokine, stromal cell-derived factor 1α (SDF-1α). Kim *et al*. demonstrated that pretreatment with EA at both Baihui (GV20) and Dazhui (GV14) acupoints for 20 min increased brain BDNF levels and upregulated the production of SDF-1α in the plasma [56]. Furthermore, some researchers indicated that EA improved neurological deficit and increase the neuroprotective activation of the PI3K/Akt pathway in the ischemic brain [57,58]. Consequently, EA-induced activation of the PI3K/Akt pathway resulted in inhibition of cerebral cell apoptosis. Moreover, EA can also increase the serum BDNF and GDNF levels, activate the PI3K pathway, and enhance the expression of anti-apoptotic signals, like Bcl-2/Bax ratio, in ischemic cerebrum. Our recent findings suggest that EA at acupoints of Quchi and Zusanli exerts neuroprotective function in ischemic stroke via activation of the PI3K/Akt pathway.

### 5.3. Memory-Deficits

Recent studies suggest that both manual acupuncture and EA can significantly improve learning memory in immobilization or chronic injection of corticosterone (CORT) rat stress models [59,60]. Furthermore, stimulation at the HT7 acupoint alone can increase BDNF mRNA and CREB in the hippocampus compared with stimulation at the Waiguan (TE5) [61]. This indicates that acupuncture can ameliorate learning memory deficits via direct activation of the neurotrophin signaling pathways, significantly reversing CORT or stress induced reduction of BDNF and CREB expression in the hippocampus. Some researchers argue that stimulation at certain acupoints can alleviate memory impairment via the regulation of BDNF resulting to neuronal cell survival and protection against neurodegeneration. Meanwhile, acupuncture can also upregulate phosphorylated CREB, which in turn, promotes BDNF expression to enhance memory [62]. Altogether, these studies suggest that acupuncture can initiate pCREB-BDNF transcription, and modulate adaptive neuronal responses required for learning and memory functions [63].

### 5.4. Inflammation

The anti-inflammatory effect of BDNF in acupuncture is mainly related to activation of the opioid and nonopioid neurotransmitters. Recent research has demonstrated that acupuncture can decrease the expression of neurotrophins (such as NGF, BDNF, and NT-3) which contribute to hypersensitivity, although it also enhances and prolongs inflammatory response. However, it has also been reported that acupuncture can down-regulate certain proinflammatory neuropeptides and neurotrophins (including BDNF and NGF) in allergic inflammation disease, and can improve clinical signs and symptoms, such as reduction in sneezing, nasal itching, rhinorrhea, and nasal congestion [64,65]. Furthermore, from this result, acupuncture might exert anti-inflammatory actions in allergic rhinitis, and not in the brain, which means the effect of acupuncture can exert different roles in different tissues, even in different pathological processes.

### 5.5. Others

Acupuncture or ear acupressure has also been shown to protect patients from blindness, and the potential mechanism may be related with the neuroprotection via regulation of NGF and BDNF and their receptors. This results in activation of the survival pathway in contrast to the stimulation of pathways related with apoptosis [66].

## 6. The Potential Mechanisms of Acupuncture with Regard to Modulation of BDNF

**Figure 1.**
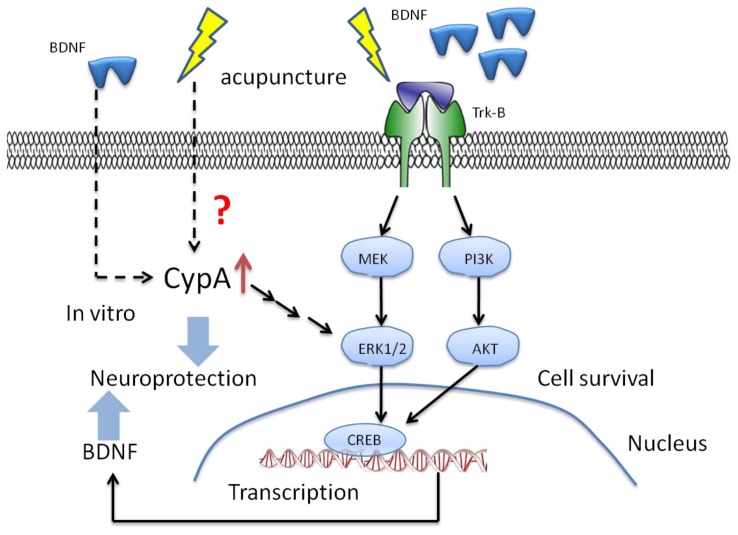
Acupuncture can increase the expression of BDNF (Brain-derived neurotrophic factor) via PI3K/Akt (Phosphatidylinositol-4,5-bisphosphate 3-kinase) and MEK/ERK1/2 (extracellular-signal-regulated kinases) signaling pathway by activating the tropomyosin receptor kinase B (TrkB), a high affinity catalytic receptor for several neurotrophins that induce the activation of survival signaling pathway. This ultimately leads to the phosphorylation and activation of the transcription factor CREB (cAMP response element-binding protein) that mediates transcription of BDNF gene expression leading to neuroprotection. In this article, we hypothesize that cyclophilin A (CypA) plays an important role in the activation of BDNF. On the contrary, BDNF may also be an important upstream factor in regulating CypA expression during acupuncture treatment. Thus, the exact mechanism between the effect of acupuncture and the expression of CypA is still unknown.

It has been reported that acupuncture can promote the expression of BDNF, and recent studies demonstrate that BDNF can protect neurons both *in vitro* and *in vivo* against various insults [35,79]. However, it has just been recently reported that acupuncture can increase the expression of BDNF and CypA in MPTP-induced mouse model of PD [74]. It was suggested that protection of DA neuronal degeneration by acupuncture was due to enhancement of CypA levels. Based on recent published studies, CypA is ubiquitously expressed in the brain [80], and is predominantly localized in neurons [81]. Moreover, although the exact functions of CypA are not yet understood, some studies suggest its contribution in neuronal differentiation and adult cortical plasticity [82,83]. Thus, if we link the increased expression levels of BDNF to CypA following EA, we can hypothesize that CypA activates BDNF and its signaling pathway via acupuncture stimulation (Figure 1). Furthermore, there are recent data which indicate that BDNF treatment induced the expression of CypA in SH-SY5Y cells [74]. Therefore, BDNF may be an important upstream factor in regulating CypA expression during acupuncture treatment. At the same time, acupuncture also plays a critical role in neuroprotection. It may work by triggering an ordered signaling pathway in which BDNF may lie upstream to other endogenous defense systems in order to protect neurons. Based on previous studies, acupuncture can increase BDNF and p-ERK1/2 protein levels in the hippocampus and the prefrontal cortex (PFC) of mice following cerebral ischemia-reperfusion injury [10,56]. *In vitro* research also confirmed that CypA stimulates ERK1/2 signal pathway in cultured cortical neurons, and protects neurons against *in vitro* oxidative and ischemic injury via activation ERK1/2 signaling pathway [84]. Collectively, one plausible explanation for the mechanism of acupuncture may be activation of the CypA-ERK pathway to upregulate BDNF expression and improve various neuronal dysfunctions [74].

Furthermore, based on some clinical studies about BDNF functions and the effects of acupuncture, we find that acupuncture can also accelerate neural regeneration in patients with some neurological disorder through neurotrophin-mediated effects. In light of evidence obtained from several animal studies, acupuncture-induced increase in endothelial BDNF exerted neuroprotection after nervous lesion. PD patients who received acupuncture for 3 months showed increased levels of BDNF in peripheral blood. However, in the spinal cord injured patients, BDNF levels were found to be decreased 48 h after acupuncture [77,78,85]. Thus, the potential function of blood BDNF in spinal cord injury remains uncertain. Early data have demonstrated that BDNF is stored in human platelets and released by physical stimulation [85]. However, the platelet number in hunman serum remained constant after acupuncture while BDNF levels decreased, although levels of BDNF-related neurotrophin NGF and other cytokines remained unchanged [78]. Although blood BDNF level has been reported to be associated with the time of acupuncture, inflammation and stress, the underlying mechanism for such relationship is still unclear.

**Figure 2.**
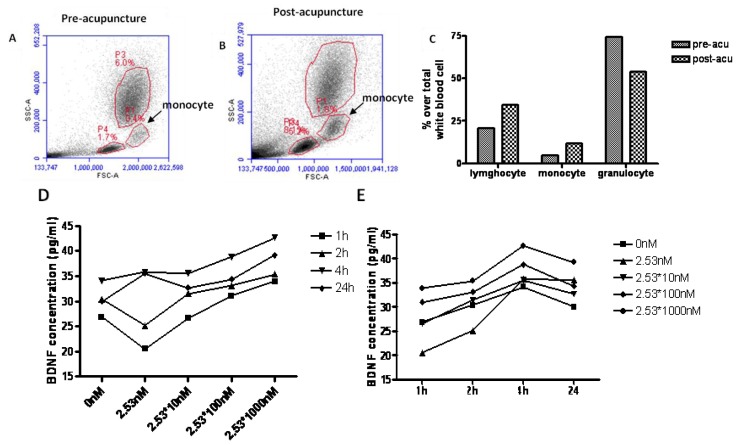
The relationship between monocytes and BDNF expression in acupuncture. (**A**) cell distribution before acupuncture; (**B**) cell distribution post acupuncture; (**C**) is the graph of data from **A** and **B**; (**D**) BDNF expression after stimulation with ATP at different concentration and (**E**) is the time course expression of BDNF level in J775 cell line stimulated with ATP; The proportion of monocyte was detected by flow cytometry before/after acupuncture. Cluster analysis of monocytes using Forward/Side scatter characteristics. The proportion of monocyte increased from 4.8%–11.8% in PBMC. The granulocyte decreased from 74.4%–53.7%. (**D**,**E**) The J774 Cell line was treated by ATP from the concentration of 2.53 nM–2.53 μM. After incubated for 24 h, the supernatant was detected by BDNF ELISA kit at 1, 2, 4, and 24 h. The result showed that ATP can induce the expression of BDNF with a dose-dependent, and reach a peak level at 4 h, and then decreased at 24 h.

On the other hand, we believe that acupuncture can also increase the expression of BDNF through the activation of monocyte. Based on the research about adenosine A1 receptors and local anti-nociceptive effects of acupuncture [86], acupuncture can increase ATP concentration, even the concentration of adenosine in local tissue. Furthermore, extracellular ATP acts as a stimulus to mediate the recruitment of monocytes in several inflammatory conditions by increasing the production of MCP-1 [87]. We hypothesize that acupuncture can modulate the activation of leucocytes (e.g., monocytes) which are known to respond to environmental simulation. Based on our recent studies, we have found that acupuncture can increase the number of monocytes in peripheral blood mononuclear cells (PBMC) in adult mice (Figure 2). Furthermore, we have cultured J774 cells, a monocyte cell line, in DMEM medium, and treated the cell with ATP at different concentrations (2.53, 2.53 × 10, 2.53 × 10^2^, 2.53 × 10^3^ nM) for 24 h. The results showed that BDNF concentration dose-dependently increased ATP concentration and that BDNF reached peak levels at 4 h. Together, these results demonstrate that ATP can stimulate the expression of BDNF in monocytes. Overall, from the preliminary experiment, we found that monocyte may serve as a bridge connecting acupuncture and the expression of BDNF.

## 7. The Potential Targets for the Effect of BDNF with Acupuncture in the Future

In view of the above-mentioned data, it is reasonable to suggest that acupuncture can prevent neuronal death and enhance neuronal survival and synaptic plasticity by activating the BDNF signaling pathway. Although the exact mechanism for such relationship is not yet defined, several studies suggest that acupoint selection is an important factor in acupuncture-induced elevation of BDNF. For example, GB34 stimulation, compared to stimulation at ST36, produced more remarkable neuroprotective effects in a PD mouse model. Stimulation at GB34 also enhanced the expression of CypA following activation of BDNF [74]. Moreover, in studies dealing with the effects acupuncture on spinal cord injury, stimulation at the CV 4 produced higher levels of BDNF and TrkB than stimulation at ST28. Focusing on the relationship between special acupoint in special models, there are some indications that acupuncture stimulation at the HT7 acupoint can significantly upregulate the expression of BDNF, than in any other acupoint on a different meridian in either spatial memory-impairment or depression-like behavioral changes model [61,67]. Together, these results indicate highly acupoint specific effects of acupuncture regardless of animal models.

## 8. Conclusions

According to the philosophical theory in Traditional Chinese medicine (TCM), acupuncture stimulation can activate the specific life energy, which is called “qi”, to regulate various organ or body functional states. BDNF and the neurotrophin family of proteins, which play a critical role in acupuncture-induced neuroprotection, will provide important insights into the nature of acupuncture. Furthermore, the therapeutic strategy of integrating acupuncture with BNDF treatment should serve as a promising treatment method in the future.

## Figures and Tables

**Table 1.** Publications of acupuncture therapy published on PUBMED in the last 10 years.

Animal research							

Reference	Experimental	Tissue	Stimulation	Time of treatment	Acupoint	Signal pathway	Result
(Kim, *et al*., 2013) [56]	cerebral ischemia in mice	cerebral cortex	Electroacupuncture 2 HZ/1 mA/20 min	EA preconditioning for 3 days	GV20/GV14		Expression of BDNF increased (after 12 h)
(Zhao, *et al*., 2013) [52]	cerebral ischemia-reperfusion injury(mouse)	hippocampus	Electroacupuncture	once daily for 7 days	BL17/GV20/BL23		BDNF mRNA expressions Up-regulated
(Chen, *et al*., 2012) [55]	cerebral ischemia/reperfusion (I/R) injury(rat)	Blood	Electroacupuncture/disperse wave of 1 and 20 Hz	30 min treated/2 or 24 h after ischemia/reperfusion	LI11/ST36	PI3K/Akt signaling pathway	increase BDNF and GDNF secretion levels in serum
(Kim, *et al*., 2012) [54]	cerebral ischaemia(rat)	ischaemic hemisphere	Electroacupuncture	once daily for 2 weeks	GV20	BDNF/trkB	increased expression of BDNF/trkB protein
(Lee, *et al*., 2012) [61]	spatial cognitive impairment induced by repeated corticosterone (CORT)(rat)	hippocampus	Manual acupuncture	once daily for 21 days	TE5/HT7		Up-regulate BDNF mRNA expressions levels
(Liang, *et al*., 2012) [50]	chronic stress-induced depression (rat)	prefrontal cortex and hippocampus	Electroacupuncture	once every other day for 28 days	GV 20/EX-HN 3/PC 6		Up-regulate BDNF mRNA and protein expression levels
(Park, *et al*., 2012) [67]	depression-like behavioral changes (rat)	prefrontal cortex (PFC)	Manual acupuncture	7 consecutive days	HT7/ST36		increased expression of BDNF protein
(Wang, *et al*., 2012) [68]	neck-incision pain rats	in the cervico-spinal cord (C1–C4)	Electroacupuncture (1–2 mA, 2 Hz/100 Hz)	30 min	LI 18/PC 6-LI 4/ST 36-GB 34	BDNF/trkB/trkA	Down-regulated for the BDNF mRNA, TrkA mRNA and TrkB mRNA
(Zhang, *et al*., 2012) [69]	spinal cord transaction between T9 and T10 (mouse)	cortex area	Electroacupuncture	once daily for 14 days	“Governor Vessel” acupoints		increased expression of BDNF protein
(Hwang, *et al*., 2010) [11]	Normal Wistar rats (13-week-old)	in the dentate gyrus of hippocampus	Electroacupuncture	once daily for 3 weeks	ST36/GV20	BDNF/CREB	increased expression of BDNF protein
(Sun, *et al*., 2010) [70]	glaucoma model in rabbits	retina	Manual acupuncture	twice a day for 4 weeks	EX-HN 7/GB 20/LR 2	BDNF/Bcl-xl	increased expression of BDNF protein
(Hua, *et al*., 2009) [71]	ovariectomized rat fracture model	fractural callus and blood samples	Manual acupuncture	once daily for 4 weeks	GB 30/ST 36/GB 34/BL 40	BDNF/trkB	increased expression of BDNF/trkB protein
(Kim, *et al*., 2009) [10]	middle cerebral artery occlusion (MCAO) rats	Cerebral ischemia area	Electroacupuncture (30 min, 2/15 Hz)	once daily for 16 days	GV20/GV14/LI11/ST36	BDNF/trkB	no significant change in BDNF
(Manni, *et al*., 2009) [72]	cognition induced by social isolation in the mouse	hypothalamus, striatum and hippocampus	Electroacupuncture (30 min, 1–4 Hz)	once daily for 4 days	ST36		Decreased expression of BDNF protein
(Wang, *et al*., 2009)[73]	spinal cord injury (rat)	spinal cord	Electroacupuncture (20 min, 1 mA, 2 Hz/15 Hz)	once daily for 10 days	CV 4/ST 28		increased expression of BDNF/trkB protein
(Jeon, *et al*., 2008) [74]	MPTP induced Parkinson’s disease mouse model	substantia nigra	manual acupuncture	once daily for 7 days	B34/SI3/BL62/ST36	BDNF/CypA	increased expression of CypA following BDNF
(Chen, *et al*., 2007) [75]	cats subjected to removal of adjacent ganglia	L6 dorsal root ganglion (DRG)	Electroacupuncture (30 min, 98 Hz)	once daily for 7 days	ST36/GB39/ST32/SP6		Up-regulate BDNF mRNA and protein expression levels
(Liang, *et al*., 2002) [76]	Parkinson’s disease rats model induced by transection of the medial forebrain bundle (MFB)	ventral midbrain/ventral tegmental area/substantia nigra	Electroacupuncture (30 min, 1–2 mA, 2/100 Hz)	once daily for 24 days	GV 14/GV 21		Up-regulate BDNF mRNA expressions levels
(Yun, *et al*., 2002) [48]	stress-induced hippocampal degeneration rats	hippocampus	Electroacupuncture (30 min, 2 Hz)	30 min (one time)	ST36		Up-regulate BDNF mRNA expressions levels

**For clinical research**							

(Xia, *et al*., 2012) [77]	Parkinson’s disease combined with depression patients	serum	electroacupuncture	3 months	GV 20/EX-HN 3/EX-HN 1/LR 3/SP 6		increased expression of BDNF(compared with that before treatment)
(Moldenhauer, *et al*., 2010) [78]	Spinal Cord Injuries	serum	Manual acupuncture	<1 h	whole-body acupuncture		Decreased expression of BDNF in 48 h after acupuncture

(The detail location of each acupoint can be refer to the website: http://www.acupuncture.com/education/points/).
